# Incorporating community-engaged research into a statewide community health worker-driven infrastructure for addressing health disparities in public health emergency

**DOI:** 10.1186/s12913-025-12859-7

**Published:** 2025-07-29

**Authors:** Azeez B. Aina, Antoniette Holt, Ashley H. Meredith, Carey Frazier, Carla Harrison, Celeste Fonseca-Tames, Fransesca Lynnet, Jasmine D. Gonzalvo, Karina Buenavides, Kourtney A.D. Byrd, Lance Boozer, Latrice Ligon, Maeve Companik, Margarita Hart, Megan Conklin, Natalia M. Rodriguez, Olivia Zarate, Olunda Hunt, Rebecca Ziolkowski, Samantha Daniels, Charleston Sanders, Shamika Crowder, Yumary Ruiz, Omolola A. Adeoye-Olatunde

**Affiliations:** 1 Department of Pharmacy Practice, Purdue University College of Pharmacy, 640 Eskenazi Ave, Indianapolis, IN 46202 USA; 2 Indiana Department of Health, Office of Minority Health, 2 N. Meridian St., Indianapolis, IN 46204 USA; 3 Indiana Community Health Workers Association, 7098, Greenwood, IN 46142 USA; 4 Department of Pharmacy Practice, Purdue University College of Pharmacy, 575 Stadium Mall Drive, West Lafayette, IN 47907 USA; 5 Department of Public Health, College of Health and Human Sciences, 812 West State Street Matthews Hall, 218, West Lafayette, IN 47907 USA

**Keywords:** Community health worker, Public health, Health equity, Social determinants of health, Epidemiology

## Abstract

**Background:**

Emergency response plans in unprecedented public health events prioritize community-based interventions for addressing health disparities. Due to their close connections and shared values with community members, positioning community health workers (CHWs) as leaders is a promising strategy for addressing health disparities. Hence, prominent public health institutions in Indiana co-developed a CHW-driven statewide infrastructure, the “Indiana Health Equity Council Community Health Worker Model” (Model) to serve as a practical framework for building a resilient and responsive public health system that strengthens the community health workforce and addresses health disparities.

**Objective:**

The objective of this study was to leverage the statewide CHW Model to generate evidence for community-centered interventions addressing health disparities across state's public health preparedness districts.

**Methods:**

A qualitative study was conducted among CHWs and CHW-affiliated organization representatives across 9 districts in Indiana. Data collection consisted of demographic surveys and audio-recorded focus groups facilitated by trained district CHWs. Verbatim transcripts of focus group recordings were coded by researchers using deductive approaches, and district-level focus group summaries were produced identifying emergent themes and proposed interventions. To assess the accuracy of findings, member checking was conducted with focus group participants. The proposed ideas to address health disparities were mapped to the Healthy People 2030 domains on Social Determinants of Health.

**Result:**

A total of 54 individuals participated in 14 focus group sessions across nine districts. Most study participants were female (*n* = 49, 90.7%) and non-Hispanic (*n* = 46, 85.2%). A high proportion worked in community-based organizations (*n* = 22, 41.5%) and performed CHW roles (*n* = 37, 69.8%). Proposed interventions for mitigating health disparities spanned health literacy programs, enculturation of communication resources, transportation services, community needs assessments, preventative health and psychosocial programs. Fifteen proposed interventions were mapped to the social and community context domain of Healthy people 2030 framework; eight aligned with healthcare access quality and two with neighborhood and built environment.

**Conclusions:**

Study findings demonstrate that leveraging the statewide Model positions CHWs to lead district efforts and generate evidence for interventions addressing health disparities across state Public Health Preparedness Districts. Future studies should assess the effectiveness of the statewide CHW Model and explore its policy implications.

**Supplementary Information:**

The online version contains supplementary material available at 10.1186/s12913-025-12859-7.

## Background

Public health emergencies are unprecedented events that disrupt several domains of the socioecological spectrum, having negative implications on health, economy, and social life [1]. Due to the magnitude of their effects and the propensity to exacerbate health disparities, public health authorities must develop robust and effective response plans that alleviate the adverse outcomes associated with these events [2]. However, the core fabric of any emergency response plan involves developing a comprehensive and response-ready community health system that disseminates resources and information, and addresses the functional needs of at-risk populations [1, 3].

Community health workers (CHWs) are among the key health professionals best positioned to align with this strategy because of their close proximity to and shared characteristics with the local communities [4, 5]. CHWs are "frontline public health workers who are trusted member of and/or has an unusually close understanding of the community served [6]." While serving as a frontline public health worker in emergencies [7–9], CHWs help to advance health equity in communities by directing their efforts toward social determinants of health (SDOH): upstream and downstream factors affecting health [10]. These can be grouped into five domains and include economic stability, education access and healthcare access, neighborhood and built environment, and social and community context. During public health emergencies, CHWs help to facilitate access to programs that align with these domains. CHWs notably connect people to food service programs and vocational training, engage in surveillance programs, and assist patients in navigating healthcare systems [8, 9].

With the increasing evidence that CHWs are effective in planning and implementing community programs addressing health disparities and barriers related to SDOH during public health emergencies, it is imperative that their pandemic-related engagements are thoroughly studied and evidence is generated for incorporating CHWs into prospective response plans [11, 12]. Public health researchers have highlighted a call-to-action for more literature on CHW programs implemented and coordinated during active crises, and such research is warranted to prospectively build workforce capacity and competencies in anticipation of future pandemics [7, 13]. Furthermore, advancing health equity through CHWs during emergencies must not only focus on their commitment to the communities during emergencies but must incorporate measures that promote professional and personal development [14].

During the COVID-19 pandemic, the Centers for Disease Control and Prevention (CDC) took a holistic approach to foster this tenet by implementing national initiatives across the United States for strengthening community resilience through training, deployment, and engagement of CHW programs addressing community needs [15]. In alignment, the Indiana Department of Health - Office of Minority Health, Indiana Community Health Workers Association, and Community Health Workforce Development Institute (Institute) at Purdue University co-developed and implemented a statewide CHW-driven model (Model) to advance health equity research, optimize the CHW workforce, and prepare communities for emergency response. This Model is responsive to the public health call-to-action by positioning CHWs at the forefront of community-engaged research to identify public health emergency-related community needs and promising evidence-based interventions for alleviating health disparities across Indiana’s Public Health Preparedness Districts.

## Methods

### Objective

The objective of this study was to leverage the statewide CHW Model to generate evidence for community-centered interventions addressing health disparities across state Public Health Preparedness Districts.

### Model description

**Fig. 1:**
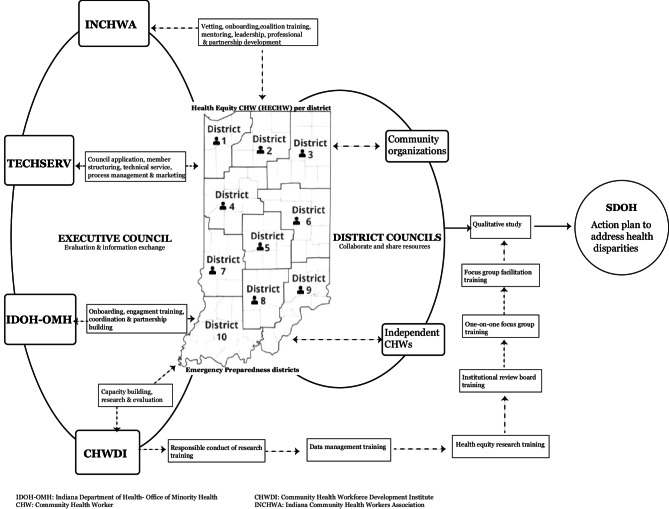
The Indiana health equity council community health worker model (Model)

The Model is a CHW-driven multipronged infrastructure comprising key and diverse entities in Indiana’s public health system focusing on health promotion, disease prevention, and health service delivery among populations experiencing health disparities. Its unique attribute is placing a CHW in each of the State’s ten public health preparedness districts to lead and create a coalition that identifies health disparities among at-risk populations and generates evidence-based interventions to address them. Each entity of the Model, including four key Executive Council organizations, up to ten district CHWs, and up to ten district councils, work in unison to contribute to the overall objectives of the Model (Fig. 1).

The Indiana Department of Health - Office of Minority Health coordinated and facilitated the employment and screening of the Community Health Worker Engagement Coordinator and 10 District CHWs while the Indiana Community Health Workers Association coached them on broad topics ranging from leadership, community engagement, professional ethics, and coalition building. The Institute focused on research training, capacity building, data analysis, and evaluation of the program. They developed a series of research trainings (Fig. 1) for District CHWs, positioning them to lead qualitative research and generate evidence-based interventions that can be implemented to address health disparities across all the public health preparedness districts. These trainings include Responsible Conduct of Research, data management, health equity, institutional review board, one-on-one focus group, and focus group facilitation. Lastly, TechServ Corporation provided technical services, process optimization and membership structuring.

### Study design

The study employed a community health worker-centered, qualitative focus group design to address study objectives. The research paradigm underpinning this study was constructivism/interpretivism, which anticipates many interpretations of reality with efforts to develop a detailed description of a particular event from those who experienced it [16]. The event herein is the COVID-19 public health emergency. To uphold research rigor and ensure reflexivity, the research team consisted of CHWs, Principal Investigator (PI), a clinical research expert (CRE), a qualitative research expert, data analysts, research assistants (RA), students, post-doc fellow, and public health and technical experts. The study was deemed exempt (category 2) from Institutional Review Board (IRB) review by Purdue’s Human Research Protection Program. The reporting guideline adopted was Standards for Reporting Qualitative Research: A Qualitative Synthesis of Recommendations (SRQR) [17].

### Conceptual framework

**Fig. 2:**
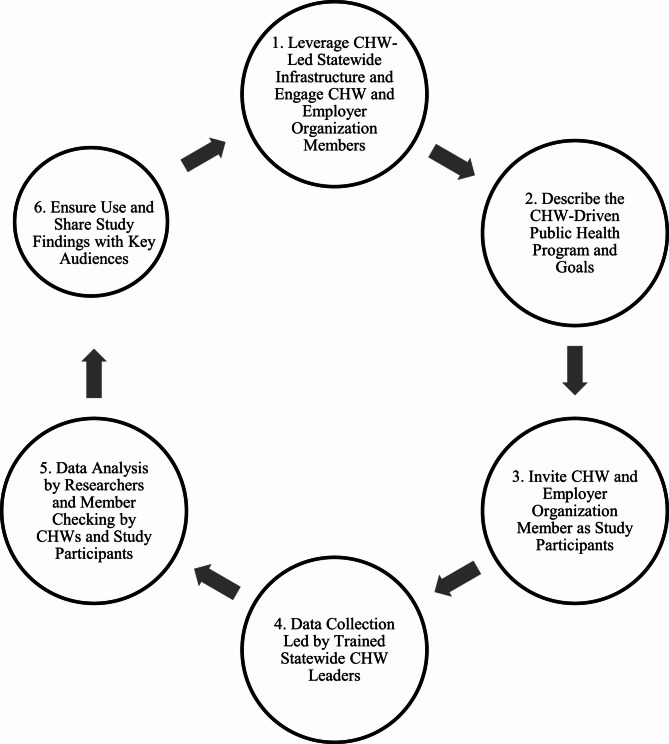
The community health worker-driven adapted CDC framework for public health program evaluation

The CDC Recommended Framework for Program Evaluation in Public Health [18] was systematically adapted to guide the processes of generating evidence in this study. The resulting adapted framework (Fig. 2) includes six steps:

1. Leverage CHW-led statewide infrastructure and engage CHW and employer organization members: the study leveraged the infrastructure to engage CHWs and employer organization members.
2. Describe the CHW-driven public health program and goals: several online/physical sessions were created to describe the program to key entities in relation to public health goals.
3. Invite CHW and employer organization member as study participants: study specifically invited CHWs and employer organizations as participants.
4. Data collection led by trained statewide CHW leaders: CHW leaders across the public health districts led the data collection process.
5. Data analysis by researchers and member checking by CHWs and study participants: data analysis was done by researchers at the Institute and member checking by study participants across districts.
6. Ensure use and share study findings with key audiences: evidence generated from study was shared with key audiences across districts.

**Fig. 3:**
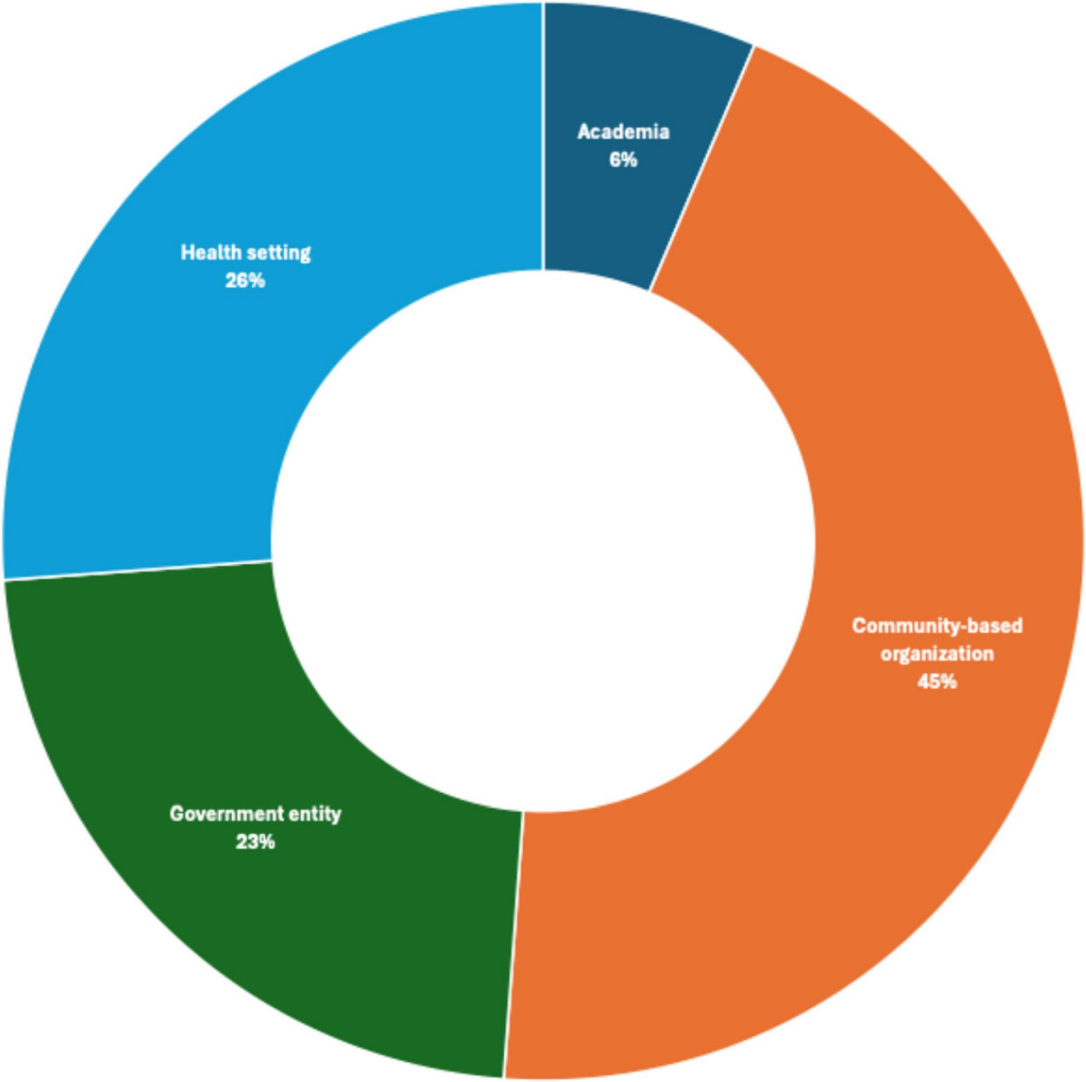
Sectors represented by organizations in Indiana's district health equity community health worker councils (*N* = 82)

District CHWs represent the Model’s core because of their bidirectional engagements with the executive and the district councils. Their central position enabled the flow of information and resources while facilitating formal and informal interactions among the network of individual CHWs and CHWs affiliated with an organization. Through their synergistic efforts, they developed multisectoral and multilevel assets that actively provide health, transportation, insurance, social, housing, education, safety, and nutrition services to community members across districts (Fig. 3).

### Setting, population & recruitment

The study was conducted in nine out of the 10 public health emergency districts in Indiana where district CHWs were hired by the state's department of health to lead pandemic-related efforts. Indiana has 10 public health preparedness districts encompassing 92 counties to facilitate the seamless distribution of resources, workforce, and capabilities among health departments, emergency agencies, hospital systems, and organizations during emergencies [19]. A purposeful sampling strategy [20] was used to recruit study participants across districts using a group characteristic sampling method [21]. A generic invitation email with a unique electronic REDCap^®^ (Research Electronic Data Capture) link was sent to each rostered council member (*N* = 240) by their district CHW to review the study information sheet (*supplementary file*) before consenting to participate in the study. The platform provided secure online support to capture data for the study with an intuitive interface, audit trails, and automated export procedures [22, 23]. An electronic questionnaire (*supplementary file*) was administered to collate participant-level demographic and professional data, and the potential date(s) and time(s) feasible to participate in the study (data described in data collection section below). Invited council members were not eligible to participate in the focus group if they did not consent to or complete the time availabile on the questionnaire. Participation was voluntary and no incentives were provided to participating council members in the study.

### Data collection

An electronic REDCap^®^ questionnaire was used to collate participant-level demographic and professional data prior to the focus group. The demographic and professional data (supplemental material) consisted of sex, race, age, ethnicity, type of organization, roles, years with the organization, whether hired for COVID and years as CHW. A focus group guide (supplemental material) containing open-ended probes was used to coordinate each session and provided ground rules for study participants and facilitators across districts. The open-ended probes were in five categories: organization’s focus/role, current efforts, facilitators, barriers, recommendations and action plans (*supplementary file*). Most focus groups were conducted within a 4-week period between May and June of 2023, except District 7, which had its session in October because of late recruitment of the district CHW. Prior to this study, district CHWs were trained in research by the Institute (Fig. 1).

A total of 240 council members were invited to participate in the study out of which 85 (35.4%) individuals consented to participate in the focus groups across 9 emergency preparedness districts. Each district had at least one focus group session, with districts 2, 3, 4, and 9 having two sessions for 14 focus groups. Each focus group was facilitated by district CHWs with each focus group lasting between 27 and 90 min. Two focus group sessions were conducted in some districts based on the schedules of eligible participants.

Each focus group session was completed via Zoom teleconferencing software [24], and was facilitated and led by the trained district CHWs with technical support from the Institute. To uphold confidentiality and anonymity, an ID number was allocated to each eligible participant and was used to maintain anonymity and increase confidentiality during focus group sessions [25]. All district CHWs had at least two practice sessions with members of the Institute before the focus groups. In addition, the Institute team verified the entry of participants prior to the focus group and ensured IRB protocols were properly upheld during sessions.

District council organization sectors were characterized using administrative, registration, research, and metadata that were culled from TechServ’s Corporation repository. The extracted document containing district organizations’ information was cleaned and verified by district CHWs while appropriating them to their respective sector.

### Data analysis

The audio was recorded verbatim and transcribed, after which the generated transcripts were cleaned, deidentified, and verified for accuracy [26]. A second accuracy check on the cleaned transcripts was done before initiating analysis. A codebook developed by the researchers was integrated to guide the coding processes and limit interpretation variability [27]. The data analysts used the codebook to deductively analyze and identify emergent themes across seven categories using a structured coding approach:

1. Organization’s primary focus and role.
2. Organization’s current efforts to address health disparities.
3. Intended population.
4. Facilitators of efforts.
5. Barriers to efforts.
6. Recommendations to address pandemic-related health disparities.
7. Efforts to inform a District Action Plan.

For districts with more than one focus group session, pairs of analysts independently coded each transcript before findings were consolidated into a district-level summary. To ensure intercoder reliability, randomly selected lines from assigned transcripts were collated and independently coded for consistency in the interpretation of quotes. Researchers iteratively met to review the emergent themes and deliberate on findings to mitigate discrepancies [28]. PI and qualitative research expert reviewed all coded transcripts and provided feedback to analysts to ensure the codebook was applied consistently [29]. Member checking [21] was facilitated by forwarding the summary of findings to council members for verification and feedback. After the summary was validated and revised as needed, the summaries were disseminated back to district councils.

Findings on efforts to inform District Action Plans were mapped to SDOH domains outlined in Healthy People 2030 [30]. Aggregated frequency/counts of areas of concentration for respective District Action Plans were computed using a quantizing technique [21] that leverages the number of recurrences of each plan. Since the action plans were geared towards addressing upstream factors affecting population health, each aggregate was mapped to Healthy People 2030 SDOH domains and objectives. Areas of concentration that aligned most with the objectives in the domain were selected because the objectives were not mutually exclusive (Table 6). Descriptive statistics was used to characterize information associated with district council organization sectors.

## Results

A total of 54 (63.5%) consented council members participated in focus groups with a minimum of 3 to a maximum of 10 participants. Most study participants were female (90.7%) and non-Hispanic (85.2%) (Table 1). A high proportion reported working in community-based organizations (41.5%), performing CHW roles (69.8%), and having 2–10 years’ experience (55.6%). A total of 82 district organizations across 9 districts were identified by district CHWs and were categorized into 4 sectors: community-based (45%), health-setting (26%), academia (6%), and government entity (23%).

**Table 1:** Demographic characteristics of focus group participants

**Characteristics**	***N***	**Response**	**Mean (SD** ^**b**^ **)**
Age	54	Age in years	47.0 (13.2)
**Characteristics**	***N***	**Response**	**n (%)**
Sex	54	Male	5 (9.3)
		Female	49 (90.7)
Race	54	Black/African American	13 (24.1)
		White	36 (66.7)
		Other^a^	5 (9.2)
Ethnicity	54	Hispanic	8 (14.8)
		Non-Hispanic	46 (85.2)
Type of Organization	53	Community-Based Organization	22 (41.5)
		Health Settings	13 (24.5)
		Academic institution	7 (13.2)
		Government Entity	11 (20.8)
Roles in organization	53	Performs CHW Role	37 (69.8)
		Certified CHW/CHW^c^	16 (30.2)
Years with organization	53	1 year or less	12 (22.6)
		2–10 years	29 (54.7)
		More than 10 years	12 (22.6)
Hired for COVID	54	Yes	4 (7.5)
		No	49 (92.5)
Years as CHW	54	1 year or less	21 (38.9)
		2–10 years	30 (55.6)
		More than 10 years	3 (5.6)

The N for different populations may not add up to 54 because of non-response

^a^Other include Biracial, American Indian, and other racial groups. They were aggregated to maintain anonymity

^b^*SD* Standard Deviation

^c^*CHW* Community Health Worker

Qualitative findings from focus groups are presented below with representative quotes.

1. Organization’s Primary Focus and Role
  Participants were affiliated with different organizations consisting of federally qualified health centers, community foundations, academic institutions, faith-based and non-profit organizations, and local health departments (Table 1). They provided a range of services to community members, which include, vaccinations, tobacco cessation, maternal and infant services and safety-net/referral programs.
  Accessibility to health and social services was central to participants’ organizations. The focus group sessions revealed that *“… [community-based organization provided] neighborhood services and one-stop shop for social service needs*” through their CHWs. The approach undertaken by these organizations varied by their mission, the population of interest, and their domains in the public health systems. While some organizations centralized their efforts to extend clinical services to populations experiencing health disparities, others committed to *“helping with strategies around healthy living and risk reduction all the way up to sort of the community level.”* The semblance of their collective efforts was in advancing health equity by directing their objectives toward upstream and downstream factors affecting community health.
2. Organization’s Current Efforts to Address Health Disparities
  During the public health emergency, organizations used different approaches to deliver efforts aimed at addressing health disparities across different populations. Although the public health emergency created fast-paced and unpredictable environmental conditions, pre-implementation efforts had to be undertaken to facilitate judicious allocation of resources. They had “*to focus on… community health assessment”* and sometimes organize *“listening sessions with organizations in the district and also lived experience of individuals”* to understand the health needs of their community before implementing any program. In addition, some organizations leveraged the existing infrastructure to implement community programs targeted at specific public health problems or health-disparity populations. A few of the community-based programs implemented by these organizations include vaccination services, health education programs, language translation services, mental health, and safety net referral services (Table 2).
  **Table 2:** Representative quotes for organizations’ current efforts

*“So not only did we*,* you know*,* try to have vaccine clinics in their communities*,* we also offered them transportation to either our facility…”* *We are continuing to educate people continuing to screen people when they first get enrolled for services* *… [what] we’re doing right now is… language services*,* improving the quality of interpretation services…”* *And working with mental health services to do mental health screenings for individuals.”*
  To maximize their input in addressing health disparities, a few organizations leveraged information and communication technologies to deliver their services to community members while prioritizing the equitable distribution of resources. They provided “*community health workers [with] tools to fight against vaccine hesitancy”* which informed the creation of an interactive “… *app where they can ask questions…”*.
3. Intended Populations
  Organizations’ populations of interest were diverse and consisted of individuals who were uninsured, living below the poverty level, aged, food insecure, retired, or individuals experiencing homelessness (Table 3).
  **Table 3:** Representative quotes for intended populations

*“… we have been working in the communities of [Northwest Indiana] to offer accessible vaccines… all free of charge*,* regardless of health insurance or even residency.”* *“… we also have the [maternal and infant support] program and… the other program for opioid use [in] pregnant people.”* *“…our goal is to reach the underserved population*,* people who are suffering from food insecurity*,* older people…”* *“We focus on individuals who are low income and the older adult population…”*
  Some study participants shared similar characteristics with the populations facing health disparities and were integral in developing specific community-based efforts. Partnering organizations aided in extending their reach to these diverse populations or communities.
4. Facilitators of Efforts
  Since many programs were implemented during the course of the public health emergency, the focus group facilitators tried to encapsulate positive efforts that influence success. Capacity building and efficient resource-sharing techniques facilitated the successful implementation of organizations’ programs through the establishment of constructive relationships with community partners to diversify and establish pathways to community resources. For instance, they “… *focused on capacity building for organizations to come together in a cohort style to learn across like six different sessions*,*…”* While these concerted efforts “*increased [organizational] capacity*,* availability of additional funding*,* specifically related to vaccine outreach efforts.”* improve workflow and productivity, it ensured synergy across organizations, especially in information sharing and the ability to “*provide services in several different counties*”. This approach extended their reach and prevented duplicity of efforts in the already-siloed public health institutions. Other factors that contributed to the positive outcomes of these programs were through the provision of culturally-tailored communication resources, partnership with legislators to enforce public health policies, marketing, vaccine education, and funding “…*our key partners in all of this, of course, have been our state representatives to help us to get the funding*..."
5. Barriers to Efforts
  Participants summarized the perceived challenges they or their organizations encountered while mitigating health disparities related to the public health emergency, which included lack of funding/resources, fatigue due to the pandemic, bureaucracy, lack of community trust, health illiteracy, siloed health system, and language barriers (Table 4).
  **Table 4:** Barriers identified by participants that contribute to health disparities

Themes	Description of Themes	Significant Statement Example
Complicated bureaucracy	Participants described different structural and systemic challenges that impede implementation of public health efforts and policies	*“[it’s] challenging for [state agency to] like directly…address the social determinants of health for these populations*,* because of the bureaucratic nature of our structure…”*
Financial constraint	Many participants indicated that financial constraints affected the extent and reach of their community-based programs	*“It’s really imperative that we acknowledge that there is a lack of funding for public health in the state.”*
Inadequate workforce capacity	Community workforce capabilities were limited and not adequate to address pandemic-related health disparities	*“…Our local health departments are overloaded. We’ve reached out… to some health departments in the area and they can’t take on any more responsibility…”*
Non-existent community partnership	The lack of pre-established community partnership diminishes community trust and reduces the engagement of community members with community-based interventions	*“So we host all these events. And sometimes we don’t have a lot of the community members show up. And I don’t know if it’s the lack [of] maybe the knowledge of the event*,* or if it’s just community members not wanting to come”*
Communication barrier	Participants highlighted that there was inadequate support system to facilitate efficient relay of relevant information to community members which was a limiting factor to healthcare access	*“We don’t have support at the federal or the local level in terms of getting that word out to our various constituencies*,* as I’ve said*,* and I don’t know if others on the call are feeling the same way.”*
Siloed public health systems	Public health institutions are disaggregated, and each unit performs different functions with no synergy among activities	*“…And different areas in the health system are doing different things. And at times*,* because we are such a large system*,* we don’t always know what others are doing…”*
Transportation access	Delivering health and social services to remote locations is pivotal to addressing health disparities and accessibility can be improved through transport	*“…at the moment is just the transportation aspect of it. Our clinic is located*,* I mean*,* and just in a low income*,* part of the community. So… the barrier of transportation is just very big for the whole community.”*
Inadequate knowledge of community resource	Awareness about available and accessible resources that can alleviate health disparities was limited during the public health emergency	*“And just people knowing what their resources really are*,* because a lot of people do not know what the resources really are. Especially the ones living out in rural areas…”*
Fatigue	Stress associated with addressing pandemic-related health disparities affected the efficiency of health care providers	*“I know people get tired of let’s go to this meeting and that meeting and zoom and*,* and all of those things*,* but I think if you can just make that little effort to maybe get out there a little bit and attend a few meetings just to see what is there…”*
  Overly complicated bureaucracy hindered the efforts to address health disparities in communities by disrupting the implementation processes of most community organizations. Most policies were not spearheaded by public health experts, and politicization of efforts and associated policies were constraints to resource dissemination and navigation. These limiting factors dictated the depth and types of community efforts that were implemented in most local communities during the public health emergency. An intrinsic characteristic of the public health system that was averse to the success of community programs was the workforce capabilities needed to deliver these programs. Participants emphasized that funding was not adequate to discharge their duties, coupled with fractured communication channels, siloed public health departments, poor transportation networks, and a lack of community trust.
6. Recommendations to Address Pandemic-related Health Disparities
  Persistent community engagement, increased diversity in workforce, efficient resource allocation, culturally-tailored translation service, preventive care, and health education were among the top recommendations for addressing pandemic-related health disparities (Table 5).
  **Table 5:** Representative quotes on recommendations for addressing health disparities

*“Advocating for higher wages for community health workers or other maybe more frontline workers… ability to offer [resources] to a wide array of folks and being able to kind of work individually…”* *“And I think we have to find a means within district to relay some of the resources available to help our community.”* *“… A good effort would be coming up with a robust list or network of mental health professionals that can help*,* I think*,* I mean*,* our community struggles with mental health…”*
  A pragmatic strategy outlined was to strengthen the CHW workforce capacity to ensure the efficient distribution of resources among “hard-to-reach populations.” They underscored some approaches that can be undertaken by public health authorities to actualize this objective, such as strengthening workforce capabilities by providing robust resources that support CHW training and a remuneration model that facilitates *“…higher wages for community health workers or other maybe more frontline workers…”*. Furthermore, participants emphasized that information outlining available resources in the community should be freely accessible and tailored to the specific population experiencing health disparities. This was reflected in the statement made by a CHW in one of the district councils: *“And I think we have to find a means within district X to relay some of the resources available to help our community.”* A participant stated that *“organizations in districts*,*… don’t continue to work in silos that we communicate a little bit more and make the effort to reach out to other ones and see what’s out there…”* Hence, programs should take cognizance of barriers to accessing health and social services during implementation while enhancing the synergies among community-based organizations for judicious resource allocation, information sharing, and collective efforts to advance health equity in communities. Prominent community strategies recommended by participants in this study included but were not limited to efforts outlined in Table 6.

**Table 6:** Summary of proposed district action plans to address health disparities

Theme	SDOH^a^ Domain^b^	Related Healthy People 2030 Objective(s)^c^	Recurring Frequency	Description of Theme	Significant Statement Example
Community needs assessment/engagement	Social and community context	Health communication	8	Engaging with community members to identify core district needs for designing effective programs	*“Maybe have a meeting of the minds with neighborhood people or other organizations that…attempted to connect… but for whatever reason did not bear fruit*,* I think if we invite the people that were all talking about wanting to serve*,* or needing to serve*,* and give them a seat at the table*,* to say what works for their neighborhood…”*
Culturally-tailored education and communication resources	Social and community context	Health communication	7	Specifically designing health education and communication resources to the values and languages of populations facing health disparities	*“…The information we provide to the client should be tailored to their culture. And not just for the x community but also for the x community in any other background…”*
Vaccination programs	Healthcare access and quality	Infectious disease	4	Community outreach to address hesitancy and provide vaccines to vulnerable populations	*“ So*,* it’s trying to figure out how to change people’s attitudes towards vaccination and toward… even just [disease] as a whole.”*
Psychosocial programs	Healthcare access and quality	Mental health and mental disorders	3	Participants suggested implementing community-based programs around mental health and substance abuse	*“… I think*,* I mean*,* our community struggles with mental health*,* not even related to COVID but adding COVID into that with people that we’re in isolation for a long time*,*…”*
Collaborate with legislators on public health challenges	Neighborhood and built environment	Health policy	1	Advocacy efforts to collaborate with legislators to make informed-decision on public health challenges	*“…like I said*,* educating the politicians so they know*,* you know that that they can go to the public health experts when it’s a public health issue”*
Transportation service	Neighborhood and built environment	Transportation	1	It was proposed that community programs should prioritize transportation access to extend access among hard-to-reach populations	*“Well*,* the transportation issue has been talked about by people being able to get the required vaccinations that they need.”*
Referral programs for SDOH	Economic stability	Economic stability- general	4	Interventions that would address upstream factors was of utmost concern in some district	*“So if I’m talking to them about*,* you know*,* [*x disease] *and health related issues*,* but they have kids at home*,* and they’re hungry*,* they’re tuning me out*,* because that’s not their primary concern…”*
Maternal health programs	Healthcare access and quality	Pregnancy and childbirth	1	While addressing mental health, a district intends to focus on maternal health	*”… I’m thinking maybe mental health surrounding infant maternal mortality with moms*,* pre-postnatal fathers*,* families…”*
Financial resources for community programs	Economic stability	Economic stability- general	1	To ensure persistent reduction in health disparities, participants advocate for continuous inflow of financial support for community-based programs.	*“…Capital F is funding*,* advocating for it? Yes*,* sometimes*,* you know*,* we don’t get what we want. But if we’re not in the face of [legislators] that I mentioned before…then we don’t exist.”*

^a^SDOH: Social Determinants of Health- Social determinants of health (SDOH) are the conditions in the environments where people are born, live, learn, work, play, worship, and age that affect a wide range of health, functioning, and quality-of-life outcomes and risks (U.S. Department of Health and Human Services. Office of Disease Prevention and Health Promotion, 2024)

^b^Domain- The aggregate/group of concepts encompassing the SDOH framework

^c^Related Healthy People 2030 Objectives: Each SDOH Domain has several and non-mutually exclusive objectives which must be attained by 2030
7. Efforts to Inform District Action Plan
  Efforts to inform the District Action Plans were community-centric interventions with the potential to reduce health disparities in each district, and included efforts aimed at community engagement, maternal health, mental health, transportation access, and vaccination. Across districts, eight interventions focused on community needs assessment and were mapped to the social and community context domain of the Healthy People 2030 framework, while four proposed efforts were specific to vaccination programs aligning with the healthcare access and quality of the framework's domain. Only one intervention was directed towards transportation service, which was operationalized as neighborhood and built environment in the framework. Table 6 depicts an overview of the identified efforts informing district action plans and how they aligned with Healthy People 2030 SDOH domains, while Fig. 4 gives visual cues of areas of concentration of these efforts across the preparedness districts.
  **Fig. 4:**
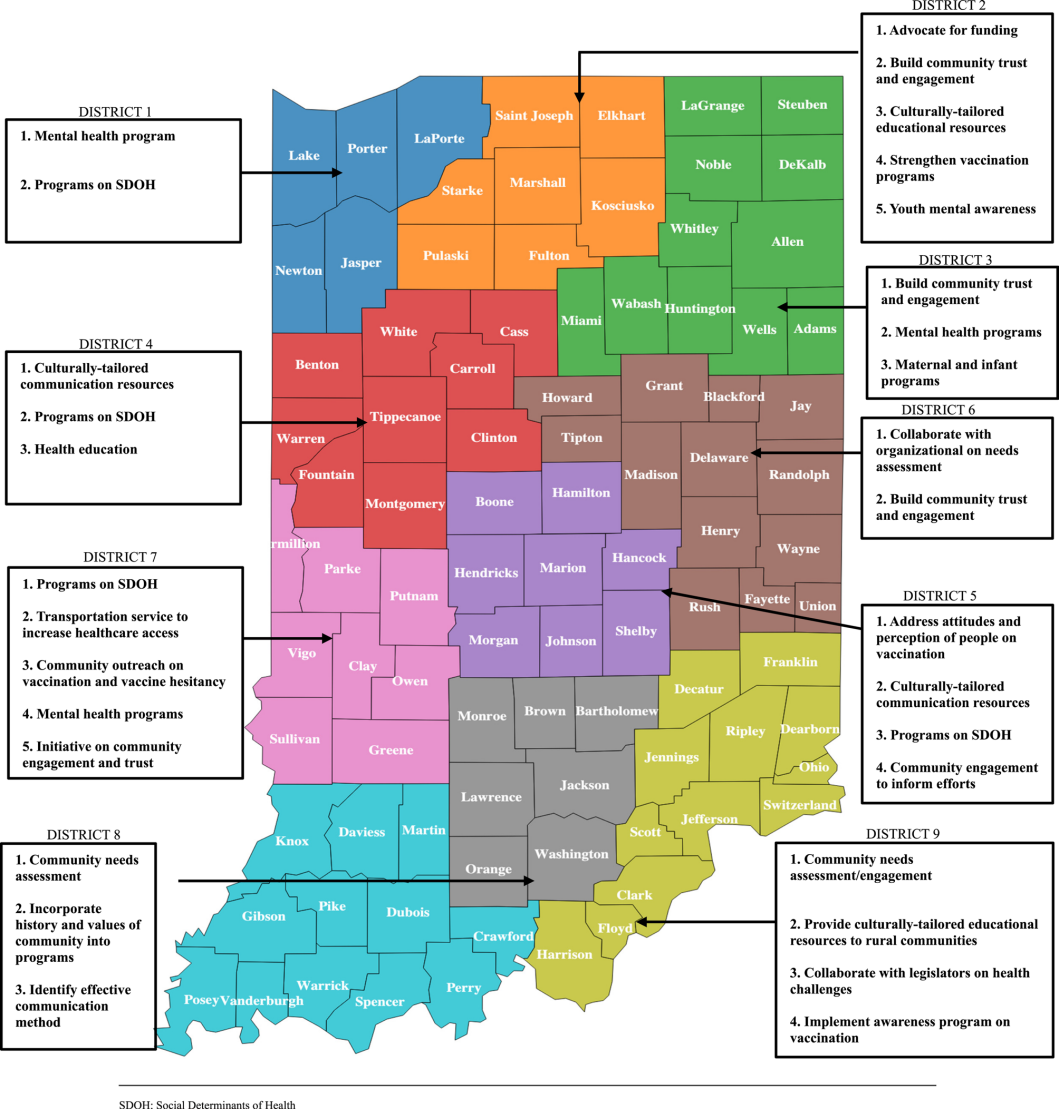
Areas of concentrated efforts that can address health disparities across Indiana's emergency preparedness districts

## Discussion

Within the purview of existing literature, this is one of the few studies to integrate community-engaged research into a statewide public health infrastructure that positioned CHWs as the frontrunner of efforts that address health disparities associated with public health emergencies. It strategically leveraged CHWs’ skills to lead and facilitate focus group research to develop interventions that will advance health equity in emergency preparedness districts. It embedded a population health approach in determining these interventions by comprehensively assessing the critical health issues and determinants necessary to improve community health [31]. As reflected in the literature, it is logical and strategic to prioritize efforts on SDOH because they impact as much as 50% of the county-level variation in health outcomes compared to 20% of clinical care in the US [32]. Hence, most of the proposed interventions by study participants in this Model were geared toward addressing SDOH.

One of the critical health issues identified in this study was the inefficiency in the bureaucratic processes of public health institutions and how this complexity derailed organizational efforts addressing health disparities. Inefficiency in workflow and communication channels can disrupt implementation strategies and ultimately reduce the overall impact of health interventions. The literature reiterated that most response plans do not integrate an intricate system that coordinates and structures society’s domains in a public health emergency [33]. Nevertheless, bureaucratic bottlenecks impeding population health can be addressed via laws and policies promulgated by policymakers given the roles the public sector plays in perpetuating health disparities [34]. Hence, participants suggested collaborating and educating policymakers in making informed decisions on public health challenges. A timely and impactful mitigation plan that addresses pandemic-related health disparities is usually initiated by policymakers, and with adequate knowledge of public health challenges, they will be able to direct and structure limited resources to the right institution. For instance, the CDC initiated a nationwide program to bolster the competencies of CHWs needed for community preparedness in the last pandemic, largely focusing on healthcare access, workforce capabilities, social support, and prevention services [15]. This initiative was informed by the American Rescue Plan Bill that was promulgated by policymakers to not just respond to the pandemic but also reinforce local public health organizations and the community workforce for public health emergencies [35]. This policy was a paradigm shift in a positive direction among the responsive efforts during the pandemic because emergency preparedness programs have been largely defunded within the past decade [36]. Therefore, participants advocated for funding sustenance in CHW and community health programs that address current healthcare challenges and prospective public health emergencies. With evidence of the critical role of CHWs in reducing health disparities and reaching underserved populations, this project offers a response strategy and sustainable recovery plan, which heralds a significant shift in public health culture, inspiring others to adopt similar approaches.

Misinformation was rife in the last pandemic, resulting in vaccine hesitancy, psychological distress, mask refusal, non-conventional medical practice, disregard for medical knowledge, and increased morbidity [37, 38]. Evidence generated from this study has the potential to help mitigate the negative influence of misinformation and inform action plans in prospective public health emergencies. Collaborative efforts from local authorities and community organizations are needed to combat the medical infodemic, and pre-established health information programs will proactively militate against reoccurrence. According to Healthy People 2030, personal and organizational health literacy have critical roles to play in advancing health equity [39]. Hence, some of the participants proposed implementing health education programs that are culturally specific to populations in their communities. This will help minimize the negative impacts of misinformation on health service delivery and ensure the right information is comprehensively delivered to diverse individuals. The district action plans for health education programs will not only focus on imparting knowledge to community members but also take cognizance of these diverse groups’ values and cultures when developing communication and learning resources.

Isolation and quarantine are strategic measures peculiar to emergency response plans to reduce the negative impact of public health emergencies and their spread [1, 40, 41]. Apart from the financial burden associated with these strategies, individuals subjected to these preventive measures are susceptible to mental harms [1, 42]. It has been revealed that several short- and long-term stressors were correlated with mental health in the last pandemic, and the prevalence was exacerbated among populations experiencing health disparities [43]. Anxiety and depression were common, but they were mostly self-reported (53%) among young adults [44]. Comprehensive mental health policies were suboptimal and overshadowed by emergency containment strategies [45]. Therefore, participants suggested integrating mental health professionals in their community programs to alleviate the long-term psychosocial impact of public health emergencies. Such interventions to protect mental health should stretch beyond acute treatment and prioritize wellness and protective behaviors [46]. Besides, evidence has shown that community interventions on mental health have had positive outcomes across the socio-ecological spectrum [47].

Finally, all the district action plans outlined in this study require support and active engagement from community members to achieve implementation outcomes. Building trust and establishing partnerships with community members, as proposed by study participants, will help advance equity, improve well-being, and increase equitable access to finite resources [48]. The anthropologic implications of these assets will provide insight into the social dynamics of health, illness, and disease transmission. Seeing how community assets, efforts, and human elements were cohesive before the crises could set us up for a better response outcome.

## Strengths and limitations

Leveraging the competencies of CHWs to facilitate the focus groups increased engagement with study participants during sessions and informed the depth and extent of the information collated. Given that study participants were widespread across nine emergency preparedness districts, the information obtained was granular and depicts the true picture of the health disparities in the state. The progression of our Model is a key aspect to consider because it outlines the program structure and comprehensively details the evaluation process while demonstrating CHWs’ concentrated efforts to address health disparities.

The differing focus group sizes, durations, and number of facilitators could impact the robustness and consistency of data collected across districts. Nevertheless, we prioritized the partnerships and trust district CHWs had with council members and their respective organizations while identifying efforts that can potentially address health disparities in their respective districts. The findings could have also been undermined by social desirability bias since most of the study participants were CHWs, and they may have given responses that subjectively favored their interests and that may not represent the true picture of the phenomenon. Since the CHWs have close connections with the community they serve, maintaining a neutral opinion during queries may be challenging, as they often share similar experiences or conditions. Additionally, their desire for improvement may influence responses, driven by the hope that assistance is on the way. Also, the generalizability of study findings is limited to a single state, but the use and description of qualitative methods and contextualized demographic information allow for transferability to other settings.

## Conclusion

Study findings demonstrated that CHWs possess the comprehensive skillsets that can be used to generate evidence aimed at addressing health disparities across Indiana’s public health emergency districts. Prominent thematic barriers among identified health disparities associated with the emergency districts consist of overly complicated bureaucracy, financial constraints, low workforce capacity, siloed public health systems, and poor transportation networks. Community interventions that have high potential for addressing health disparities and advancing health equity in these districts include community needs assessment/engagement, vaccination programs, maternal and mental health programs, free transportation services, and culturally-tailored health education resources. Future studies should assess the effectiveness of the statewide CHW Model and explore its policy implications.

## Study implications

The findings from this study have significant policy and practical implications in addressing health disparities across emergency districts in the State of Indiana. The study is currently in phase 2 where the proposed district action plans are being implemented over a 9-month period with active participation from several community and state-level public stakeholders. Data generated from this phase will inform the evaluation processes and future studies that will emanate from this project. Also, the CHW workforce is burgeoning in Indiana with increasing demand for CHW-related services. However, the state of Indiana is still lagging in the vertical integration of these professionals into its public health systems, with only one out of 90 counties implementing active billing codes for CHW services. Evidence from this study will influence the state’s policies that can positively transform the landscape of CHW professionals while promoting their adoption.

## Supplementary Information

Supplementary Material 1.

Supplementary Material 2.

Supplementary Material 3.

## Data Availability

All focus group recordings and datasets are available from the corresponding author upon reasonable request. The study information sheet, focus group guide and questionnaire used for collating high-level participant information have been attached as supplementary files. We would also like to declare that the email addresses of most of the co-authors have changed due to the ongoing layover and funding restrictions associated with the project.
