# Supplementary material for: Incorporating community-engaged research into a statewide community health worker-driven infrastructure for addressing health disparities in public health emergency
Source: BMC Health Serv Res. 2025 Jul 29;25:991. doi: 10.1186/s12913-025-12859-7 (PMC12309110; doi:10.1186/s12913-025-12859-7)
Supplement: Supplementary file 1 — Supplementary Material 1. [file 12913_2025_12859_MOESM1_ESM.docx]

**Welcome! Thank you for your interest in learning more about this study and how you can get involved.**

**Please carefully read the study information sheet below. Then, you will be asked to specify whether or not you would like to participate in the study. If you agree to participate, you will also be prompted to fill out a short questionnaire. Finally, don't forget to hit the submit button.**

If you have questions, comments or concerns about this study, you can talk to one of the researchers. Please contact Principal Investigator, Dr. Omolola Adeoye-Olatunde at 317-880-5417 or [adeoyeo@purdue.edu](mailto:adeoyeo@purdue.edu) at any point. You may also contact Program Manager Dr. Kourtney Byrd at 773-757-3441 or [byrdka@purdue.edu.](mailto:byrdka@purdue.edu)

**Section 1 Study Information Sheet**

**STUDY INFORMATION SHEET**

### Phase 1: Indiana Health Equity Council (HEC) Community Health Worker (CHW) Model

IRB Protocol Number: IRB-2022-882

Omolola (Lola) Adeoye-Olatunde, PharmD, MS, Assistant Professor

Department of Pharmacy Practice

Community Health Workforce Development Institute (CHWDI)

Purdue University

# **What is the purpose of this study?**

You are being invited to participate in a Focus Group, because you are an Indiana Health Equity Council (HEC) member. You should read the information below, and ask questions about anything you do not understand before proceeding. The purpose of this phase 1 study is to gather insight into what is going well, what can be improved, and what needs to happen regarding addressing COVID-19-related health disparities in your district to form your HEC District Action Plan (to be developed and evaluated in phase 2).

# **What will I do if I choose to be in this study?**

If you agree to participate, you will participate in a moderator-led focus group for this research project. We are conducting one focus group with each of Indiana’s ten HECs. A focus group ordinarily means group interview. We are conducting focus groups to gain a deeper understanding of council member organizations' needs to better address COVID-19-related health disparities in their community and synergize efforts across council member organizations. The information gathered from the focus group will generate ideas for a 9-month District Action Plan to be developed and evaluated in phase 2 of this study.

If you agree to participate, you will be asked to do the following:

- Provide some background information, such as your age and organizational affiliation, on the next few pages after you’ve read all of the study information and before participating in the focus group.
- Participate in one, 2-hour focus group alongside any other members of your district HEC who agree to participate. This focus group will be led by either your district HECHW or CHW Community Engagement Coordinator, Shamika Crowder, who are all trained in focus group methods. They will ask you questions about how your organization and district have addressed COVID-19 and your opinions on how it has been addressed. Focus groups will be conducted in person/virtually and will be recorded and transcribed. Focus group transcripts will be de-identified, meaning that the notes cannot be linked to any specific participant. De-identified handwritten or typed notes may be recorded by one of the researchers during the focus group.

Examples of the types of questions that might be asked during the focus groups include:

- *Currently, what efforts are your organizations engaged in to address COVID-19-related health disparities in your district?*
- *How do these efforts overlap or differ from other council members’ efforts?*
- *What is going well with your organization’s current efforts?*
- *How can these current organizational efforts be improved?*
- *What efforts should this council focus on to addressCOVID-19-related health disparities in your district?*

# **How long will I be in the study?**

You can expect to spend about 5-10 minutes to complete the background information questionnaire and a maximum of 2 hours at the moderator-led focus group (the exact duration of the focus group will be included in a calendar invite that you will receive via email). Researchers will follow up with you afterwards on an as needed basis. For instance, we might reach out to you to clarify one of your comments from the focus group. Researchers might also reach out to you in the future to see if you are interested in participating in phase 2 of this study or other related studies.

# **What are the possible risks or discomforts?**

# There are no significant risks for those who participate in this study. You may feel uncomfortable answering questions regarding the way COVID-19 has been addressed in your district. If you feel uncomfortable or prefer not to answer a question for any reason, you can choose to remain silent and not answer the question, or you can tell the researcher that you would like to skip that question. Also, if you feel uncomfortable or decide not to participate for any reason, you may leave the focus group at any time with no repercussions.

# There is a slight risk to your reputation if confidentiality is breached in that it might become known how you have addressed COVID-19 in your district or how you feel about the way it has been addressed. Study procedures such as storing data in a secure computer folder will minimize this risk. Only the research team will have access to these data. The time burden is expected to be minimal, mainly the time to participate in the focus group.

# **Are there any potential benefits?**

# It is not expected that you will gain any immediate personal benefit from participating in this study. However, it may give you the opportunity to voice your opinion and ideas about how COVID-19 and related health disparities are being addressed in your district. Over time, the study results might improve the way COVID-19 and related health disparities are being addressed in your district as a result of participating in the study.

# Information gathered from the focus groups will be used for research purposes and may be shared with Purdue University, the Indiana Department of Health (IDOH), the Indiana Community Health Workers Association (INCHWA), and others who are in a position to improve the HECHW model for future use. It is anticipated that this study will help inform future use of the HECHW model within Indiana and possibly other settings.

**What alternatives are available?**

You do not have to participate in the study if you do not want to. There will be no repercussions or consequences if you choose not to participate. You may leave the focus group at any time if you feel uncomfortable or for any other reason.

**Will I receive payment or other incentive?**

There will be no direct payment, reimbursement, or incentive for participation in this focus group.

**Conflict of Interest Disclosure:**

The investigators of this study have nothing to disclose.

# **Will information about me and my participation be kept confidential?**

# The project's research records may be reviewed by departments at Purdue University responsible for regulatory and research oversight. The research team will make every possible effort to keep your personal information confidential. However, we cannot guarantee absolute confidentiality. In accordance with the law, confidentiality cannot be guaranteed if the researcher becomes aware that you may be a danger to yourself or to others, or becomes aware that acts of child abuse or elder abuse may have occurred. Your identity will be held in confidence in reports in which the study may be published.

# **What are my rights if I take part in this study?**

Your participation in this study is voluntary. You may choose not to participate or, if you agree to participate, you can withdraw your participation at any time without penalty or loss of benefits to which you are otherwise entitled.

**Who can I contact if I have questions about the study?**

If you have questions, comments or concerns about this study, you can talk to one of the researchers. Please contact Principal Investigator, Dr. Omolola Adeoye-Olatunde at 317-880-5417 or [adeoyeo@purdue.edu](mailto:adeoyeo@purdue.edu) at any point. You may also contact Program Manager Dr. Kourtney Byrd at 773-757-3441 or [byrdka@purdue.edu.](mailto:byrdka@purdue.edu)

If you have questions about your rights while taking part in the study or have concerns about the treatment of research participants, please call the Human Research Protection Program at (765) 494-5942, email ([irb@purdue.edu](mailto:irb@purdue.edu)) or write to:

Human Research Protection Program - Purdue University

Ernest C. Young Hall, Room 1032

155 S. Grant St.,

West Lafayette, IN 47907-2114

**Section 2 Consent**

Please indicate whether or not you consent to participate in this study.

 I consent to participate in this study (proceeds to section 3)

 I DO NOT consent to participate in this study (proceeds to Thank you statement)

 I would like to talk to a researcher about this study before making my decision

We understand you want to talk to a researcher about this study. Please contact Principal Investigator, Dr. Omolola Adeoye-Olatunde at 317-880-5417 or adeoyeo@purdue.edu at any point. You may also contact Program Manager Dr. Kourtney Byrd at 773-757-3441 or byrdka@purdue.edu.
