# Supplementary material for: Incorporating community-engaged research into a statewide community health worker-driven infrastructure for addressing health disparities in public health emergency
Source: BMC Health Serv Res. 2025 Jul 29;25:991. doi: 10.1186/s12913-025-12859-7 (PMC12309110; doi:10.1186/s12913-025-12859-7)
Supplement: Supplementary file 2 — Supplementary Material 2. [file 12913_2025_12859_MOESM2_ESM.docx]

**Section 3 Demographics**

What is your age: ____________

Which gender do you most identify with?

 Female

 Male

 Non-binary or gender nonconforming

 Prefer to Self-Describe: ________________________

 Prefer not to answer

What is your race (select all that apply)?

 American Indian or Alaska Native

 Asian

 Native Hawaiian or Pacific Islander

 Black or African American

 White

 Prefer to Self-Describe: _______________________

 Prefer not to answer

What is your ethnicity:

 Hispanic or Latino/a

 Not Hispanic or Latino/a

 Prefer to Self-Describe: _______________________

 Prefer not to answer

Which most accurately describes the type of organization you are representing:

 Community-Based Organization

 Health Setting (e.g., health system, independent clinic etc.,)

 Local government entity (e.g., county health department)

 I am not representing an organization. I joined the council as an individual

 Something Else:_____________________

What is your organizational role:

 Certified Community Health Worker

 Community Health Worker

 Something Else:__________________

Where you hired specifically for COVID-19 related work?

 Yes

 No

 Prefer to Self-Describe:_________________

What is your length of involvement with the organization you are representing:

 1 year or less

 2-4 years

 5-10 years

 11-15 years

 16-20 years

 More than 20 years

How long have you been performing the role of a CHW?

 1 year or less

 2-4 years

 5-10 years

 11-15 years

 16-20 years

 More than 20 years

Thank you for completing this questionnaire! Your response has been recorded.
