# Supplementary material for: Incorporating community-engaged research into a statewide community health worker-driven infrastructure for addressing health disparities in public health emergency
Source: BMC Health Serv Res. 2025 Jul 29;25:991. doi: 10.1186/s12913-025-12859-7 (PMC12309110; doi:10.1186/s12913-025-12859-7)
Supplement: Supplementary file 3 — Supplementary Material 3. [file 12913_2025_12859_MOESM3_ESM.docx]

**Focus Group Guide**

**Welcome** and thank you for taking part in this focus group. We are interested in your views regarding what is going well, what can be improved, and what opportunities needs to be explored regarding how to address COVID-19-related health disparities in your district. We realize you are busy and appreciate your time.

**Ground rules**

1. (For virtual) For confidentiality purposes, we have disabled cameras and changed your screennames to match your assigned number. Please take a moment now to make sure your camera is off and your screenname matches the number you were just assigned. If you’re having any issues, please let <<<Purdue team member name>>> know.
2. (For virtual) Please use the raise hand function on Microsoft Teams to indicate that you would like to speak.
3. (For virtual) You may not make any comments using Microsoft Team’s chat function, all discussion must be verbal.
4. Please say your assigned participant number prior to speaking each time you share.
5. If you agree with a response that has already been captured, you do not need to restate their response, but we encourage you to briefly voice your agreement (ex. “This is participant 3, and I agree with what participant 7 just said”).
6. You can give perspectives from your own experience or the experience of other co-workers you know, but please do not reveal the name or any identifying characteristics about any other person’s perspectives.
7. You may speak in any order, but only one person should speak at a time.
8. Wait until a person has completely finished their comment before jumping in.
9. We have a lot we need to discuss in a relatively small amount of time. Please keep your answers brief and precise, and be aware that I may have to cut off discussion on a given subject so that we can move on to the next topic
10. You do not have to agree with the views of other people in the group, but be respectful when you disagree with their opinions.
11. There are NO right or wrong answers.

Do you have any questions before we begin and start recording?

**VERBAL RECORDING LABEL**

**Facilitator:** This is focus group number ___ taking place in district # on MM/DD/YYYY being facilitated by <<<Name>>>.

**ICE BREAKER (10 MINUTES)**

**Facilitator:** To start things off, let’s start with an ice breaker. Can you each tell me a little about your organization? What is its primary focus?

1. What is your role?

**CURRENT EFFORTS (15 MINUTES)**

1. What does your organization currently do to address COVID-19-related health disparities in your district?
   1. Who do you, or are trying to reach and why?
   2. How have you gone about this?
2. How do your efforts overlap or differ from the other council member’s efforts shared thus far?

**Facilitator:** Now let’s discuss what you specifically think is going well with your organization’s current efforts to address COVID-19-related health disparities in your district.

**WHAT IS GOING WELL? [FACILITATORS] (20 MINUTES)**

1. What is going well with your organization’s current efforts?
   1. What are key things, resources, partners, etc., that your organization has that you think have aided this success?

**Facilitator:** Now let’s discuss specifically what you think can be improved with your organization’s current efforts in addressing COVID-19-related health disparities in your district.

**WHAT CAN BE IMPROVED? [BARRIERS] (20 MINUTES)**

1. What current organizational efforts can be improved?
   1. What are key things, resources, partners etc. that your organization is lacking making it hard to have the success you would like to have?

**Facilitator:** Now that you’ve heard everyone’s experiences and areas of need, let’s wrap-up with discussing specific areas and ways you think this council can work together to address COVID-19-related health disparities in your district. This will be what guide your Council 9-month DAP.

**WHAT NEEDS TO HAPPEN? [RECOMMENDATIONS for DAP] (30 MINUTES)**

1. What efforts should this council focus on to address COVID-19-related health disparities in your district?
   1. Please elaborate?
   2. How?
   3. I’m going to pull up a grid to help us prioritize this effort (share screen and pull up the grid). Would you say this effort is:
      1. high impact and feasible to implement over 9 months with council resources
      2. High impact and not feasible to implement over 9 months with council resources
      3. Low impact and highly feasible to implement over 9 months with council resources
      4. Low impact and not feasible to implement over 9 months with council resources
   4. Tell me some more about went into rating the effort this way?
   5. What resources would be needed to make it feasible?

**CONCLUDING QUESTION (5 MINUTES)**

1. Of all the things we’ve discussed today, what would you say are the most important to consider when addressing COVID-19 related health disparities in your district?

**Conclusion:**

Thank you for participating in this focus group! We hope that you have found this discussion interesting. The experiences you shared will be a valuable asset to this initiative and are expected to guide future council plans. Have a good afternoon/evening!
